# Water stress and recovery dynamics of physiological function and growth in juvenile *Pinus radiata*

**DOI:** 10.1093/treephys/tpag051

**Published:** 2026-06-08

**Authors:** Dejan Firm, Damien Sellier, Warren Yorston

**Affiliations:** New Zealand Institute for Bioeconomy Science, Titokorangi Drive, Private Bag 3020, Rotorua 3046, New Zealand; New Zealand Institute for Bioeconomy Science, Titokorangi Drive, Private Bag 3020, Rotorua 3046, New Zealand; New Zealand Institute for Bioeconomy Science, Titokorangi Drive, Private Bag 3020, Rotorua 3046, New Zealand

**Keywords:** cambial growth, carbon assimilation, drought recovery, gas exchange, isohydric

## Abstract

Forests worldwide face increasingly frequent, prolonged and severe droughts driven by climate change, causing widespread tree dieback and productivity losses. Yet predicting how trees recover their water–carbon balance and growth after non-lethal drought remains challenging. To address this, we subjected juvenile *Pinus radiata* D.Don—a drought-tolerant, strongly isohydric conifer—to moderate (14 weeks) and extended (20 weeks) dry-down periods. We continuously monitored stem radial growth and water reserves for over 6 months with high-resolution dendrometers, alongside weekly measurements of leaf gas exchange, capturing fine-scale dynamics of the water–carbon balance and growth during stress and recovery. We recorded a complete recovery of leaf function and stem growth in all plants after drought release, but the recovery rate depended on drought duration: plants under prolonged stress recovered more slowly. Stomatal conductance returned to control levels after $\sim$1.5 and 2.5 weeks following moderate and extended drought, respectively, whereas net $\mathrm{CO_{2}}$ assimilation recovered within $\sim$1.5 weeks regardless of drought duration. In contrast, cambial activity resumed rapidly, within a few days up to a week, as soon as stem water reserves were refilled. Growth recovery was rapid even in plants that experienced very low water potentials and nearly 2 months in a neutral or negative carbon balance state. Wood formation resumed ahead of photosynthesis recovery, reflecting a decoupling between carbon source and sink processes. Although drought reduced total radial growth, aboveground biomass gain in stressed plants remained comparable to that of well-watered controls, even for those with a growing period halved because of drought. This high degree of growth resilience arose through compensatory growth, with post-stress growth rates 1.4–2.4 times higher than pre-stress rates. These findings provide new insights into drought responses and recovery in a conifer with conservative water regulation and will improve model predictions of juvenile-tree resilience under future climates.

## Introduction

Climate warming and intensifying drought pose severe threats to forests globally (Brodribb et al. 2020, Novick et al. 2024). Recent analyses report rising tree mortality rates worldwide and hotspots of forest dieback tied to more frequent hotter droughts (Hammond et al. 2022). Moreover, satellite- and tree-ring-based studies reveal that many tropical, arid and temperate forests have experienced declining ecosystem resilience under increasing water limitation, coinciding with pervasive declines in productivity (Anderegg et al. 2015, Forzieri et al. 2022, Zheng et al. 2023). How soil water deficit and elevated evaporative demand—a signature of warming—impair hydraulic functioning, carbon assimilation and ultimately growth, is well documented (Tardieu et al. 2011, Park Williams et al. 2013, Körner, 2015, Krejza et al. 2021, Novick et al. 2024). The available evidence underscores that climate-driven stressors are already weakening forest carbon uptake and growth. With climate projections pointing to more frequent and severe droughts, it is critical to understand the mechanisms by which different tree species and forest types will cope under future conditions.

Trees regulate water loss and carbon gain in concert, but drought can decouple carbon source and sink processes (Muller et al. 2011, Fatichi et al. 2014, Körner, 2015, Cabon et al. 2022), leading to a transition from source to sink growth limitation (Cabon et al. 2024). Beyond the biophysical limits of turgor-driven cell division and expansion, this decoupling also reflects shifts in carbon allocation priorities under stress, aimed at preserving vital physiological functions. For example, trees may preferentially allocate carbon to reserves (Muller et al. 2011, Sala et al. 2012, Rehschuh and Ruehr, 2022). Stomatal regulation is the primary mechanism through which trees control their water status, and the drying of the soil and/or atmosphere typically induces partial stomatal closure to protect against xylem hydraulic dysfunction (Brodribb and McAdam, 2013, Martin-StPaul et al. 2017, Sharma et al. 2025). Because stomatal closure reduces transpiration more strongly than $\mathrm{CO_{2}}$ assimilation, this results in an increase in tree water-use efficiency (Beer et al. 2009, Yi et al. 2019, Zhang  et al.  2019), which has an ameliorative effect on the whole-tree water and carbon balance under stress. However, once the leaf water potential falls below a critical threshold, the stomata close almost completely and carbon assimilation ceases. Under those conditions, trees depend on stored non-structural carbohydrates (NSCs) to sustain metabolism, respiration and any remaining growth (McDowell et al. 2008, Mitchell et al. 2013, Hartmann and Trumbore, 2016). In parallel, growth rates (e.g., cambial activity) decline and eventually halt under drought as cell turgor is lost (Lockhart, 1965, Tardieu et al. 2011, Körner, 2015). As growth is usually affected earlier and to a larger extent than carbon assimilation and metabolism, the concentration of NSCs in plant organs generally increases (Muller et al. 2011, O’Brien et al. 2015), at least under short to moderately long periods of water stress.

Previous studies have shown that the degree of coordination between carbon source and sink activity during water stress and post-stress recovery strongly depends on stress severity and duration (Brodribb and Cochard, 2009, Ruehr et al. 2019, Li et al. 2021). Mild to moderate water stress often allows for the rapid recovery of gas exchange (Blackman et al. 2009, Brodribb and Cochard, 2009, Li et al. 2016), but the actual rate of recovery depends on the mechanisms of stomatal regulation, which are species-specific and influenced by differences in abscisic acid dynamics (Blackman et al. 2009, Brodribb and McAdam, 2013). Similarly, growth can recover quickly if the stress has not caused functional damage to the hydraulic system of the plant. In contrast, in the case of severe stress, the restoration of hydraulic function, carbon uptake and growth processes can require the costly repair of tissues using stored NSCs (McDowell et al. 2008, Ruehr et al. 2019, Hikino et al. 2022, Rehschuh et al. 2022). Notably, severe droughts can cause substantial xylem damage, requiring recovery through refilling embolized conduits (Klein et al. 2018, Trifilò et al. 2019) or producing new ones after stress release (Brodribb et al. 2010, Hammond et al. 2019, Gauthey et al. 2022). Furthermore, under prolonged drought stress some species that have a very conservative water regulation behaviour can experience a significant depletion of NSC reserves (Mitchell et al. 2013), which can greatly reduce the ability to recover from sublethal stress once critical carbon thresholds are exceeded. In summary, drought severity and duration strongly influence recovery trajectories. Conceptual models suggest that as stress severity increases, the carbon cost of recovery rises sharply (Trugman et al. 2018, Ruehr et al. 2019). This has important implications for modelling forest responses to future droughts. For example, after an extreme drought, global and regional tree-ring studies reveal many sites with incomplete stem-growth recovery persisting for years (Galiano et al. 2011, Anderegg et al. 2015). Conversely, single mild drought events are unlikely to have any lasting impact.

Juvenile trees can be particularly vulnerable to drought and elevated temperatures, compared with mature trees, which often have significant acclimation abilities (Padilla and Pugnaire, 2007, Van Mantgem and Stephenson, 2007, Will et al. 2013, Davis et al. 2019). Young plants have relatively shallow root systems that depend on the topsoil moisture. Furthermore, they have much smaller stem water and carbon reserve pools. Non-structural carbohydrate reserves are notably small compared with mature trees (Hoch et al. 2003, Niinemets, 2010). Although NSC reserve requirements scale with size and juvenile trees do not need excessively large carbon reserves (Sala et al. 2012), they still rely on those reserves to buffer carbon deficits during and after stress (Mitchell et al. 2013, O’Brien et al. 2015). For example, tropical tree seedlings pre-enriched with NSCs maintained higher water potentials and hydraulic function during drought, delaying mortality (O’Brien et al. 2014). By contrast, depleted seedlings experienced lower survival. Thus, reductions in carbon assimilation, even from a mild drought event, can disproportionately constrain survival and post-stress growth in the juvenile phase (Niinemets, 2010, Li et al. 2021, Gómez-Gallego et al. 2025). Although prolonged water stress can have legacy effects on growth and induce delayed mortality, some studies instead document a full recovery and increased annual growth (Li et al. 2016, O’Brien et al. 2017). These outcomes are often species- and life-stage-specific (Lloret et al. 2011, O’Brien et al. 2017, Song et al. 2022, Hesse et al. 2023), as some species can exhibit compensatory growth during the recovery phase after drought. Unfortunately, few studies provide high-resolution, continuous data on how gas exchange and cambial activity covary during and after drought in young trees (Mitchell et al. 2014, O’Brien et al. 2015, Blackman et al. 2019, Rehschuh and Ruehr, 2022) and how different stress intensities shape the recovery pattern. Capturing fine-scale temporal patterns of the water–carbon balance and radial growth is needed to link assimilation with stem tissue formation (e.g., Steppe et al. 2015, Blackman et al. 2019). This in turn is essential for developing a mechanistic understanding of post-stress recovery and growth dynamics.

In this study, we experimentally investigate these processes in juvenile *Pinus radiata* D. Don trees, a drought-tolerant conifer widely grown in fast-rotation plantations. The species has a water-regulating behaviour that can be characterized as strongly isohydric (Brodribb and McAdam, 2013, Blackman et al. 2019, Sharma et al. 2025). The stomata close rapidly under water stress to protect hydraulic integrity, at the cost of reduced carbon gain. The species also exhibits other traits associated with a drought avoidance strategy, such as needles that are highly resistant to desiccation (Blackman et al. 2019). We grew potted plants under ambient glasshouse conditions and imposed two levels of prolonged soil drought by withholding soil irrigation for 14 and 20 weeks. Our overall objective was to determine how a prolonged soil moisture deficit and its relief affect the coordination between carbon uptake and growth in juvenile trees. We specifically aimed to investigate: (i) how the duration of water stress affects stomatal conductance and photosynthesis over time, (ii) the extent to which changes in gas exchange during water stress translate into reductions in cambial activity, (iii) the coordination between the resumption of carbon assimilation and cambial activity after drought release, (iv) whether there is a relationship between the magnitude of water stress and the recovery rate and (v) the net effect of drought intensity on total stem radial increment and height growth over the season.

## Materials and methods

### Plant material and growing conditions

**Figure 1:**
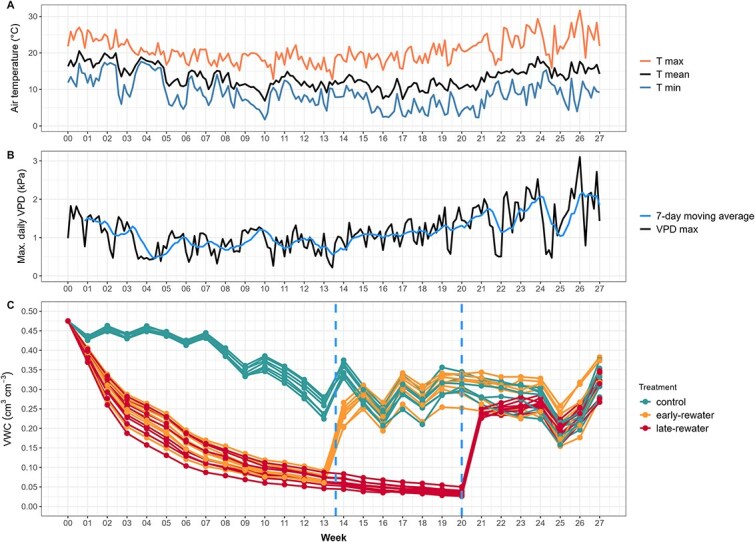
Daily air temperature (A) and maximum vapour pressure deficit (B) in the glasshouse over the 28-week period. (C) Temporal pattern of soil volumetric water content changes for individual pots with plants (*n* = 23). Dashed vertical lines indicate the first rewatering of plants in the early- (middle of week 14) and late-rewater (beginning of week 21) treatments, respectively.

The study was carried out using 24 juvenile *P. radiata* trees. Two genotypes (G1 and G2) with no prior known differences in growth performance or hydraulic traits were tested to control for potential genetic variability. Each genotype was equally represented by 12 plants. Each plant was propagated by organogenesis, i.e., stem cuttings, from a mother plant originally propagated by somatic embryogenesis. This ensured that genetic variation within a genotype was minimal. Both genotypes are representative of those currently planted in New Zealand timber production forests. All plants were 18 months old at the beginning of the experiment. Stem height ($\overline{x}$  $\pm$ SD: 610 $\pm$ 78 mm) and diameter (9 $\pm$ 1 mm) were similar for all plants. They were potted in 9-L well-draining pots filled with a homogeneous potting mixture containing 35% composted pine bark fines, 35% coconut fibre, 30% granulated pine bark (3–6 mm) with added controlled-release fertilizers and micronutrients. The plants were placed in a glasshouse facility at the Scion nursery in Rotorua, New Zealand (38.16S, 176.27E, 300 m a.s.l.) for the duration of the experiment. The environment was sheltered from wind and rain but without active regulation of air temperature and humidity (Figure 1A and B). The daytime air temperature in the glasshouse varied between 9.3 $^{\circ }$C and 24.6 $^{\circ }$C (16.1 $\pm$ 3.2 $^{\circ }$C), and the relative humidity ranged between 44% and 89% (68 $\pm$ 10%). The mean air temperature in the glasshouse was 4.4 $^{\circ }$C higher than the outdoor air temperature ($T_{a}$) between April and September.

### Experimental design

The plants were individually labelled with a unique identifier consisting of genotype and specimen number. They were randomly assigned to three groups of eight samples each: control (C), early-rewater (EW, ‘moderate drought’) and late-rewater (LW, ‘long drought’). Each group had an equal representation of genotypes, i.e., four samples of each genotype by group. A subset of 12 plants—four by group—was selected for gas exchange measurements. The plants were randomly placed on a nursery table independently of their group. The experiment started on 31 March 2023. All plants were initially watered so that the soil was near field capacity. Subsequently, the C plants were watered throughout the duration of the experiment to ensure soil water content remained above $\sim$20%, i.e., every 3 weeks on average (Figure 1C). For EW plants, soil watering was withheld until 10 July, i.e., the dry-down period was $\sim$13.5 weeks long. For LW plants, irrigation was withheld until 24 August, i.e., the dry-down lasted for 20 weeks. The end of the dry-down period for the EW group was set to the time when full daytime stomatal closure was reached, while the drying-down was continued for an additional seven weeks after that time in the LW group. Following the end of each group’s dry-down period, plants were fully rewatered and thereafter irrigated on the same schedule as control plants to maintain comparable, non-limiting soil water content until the end of the experiment. We concluded the experiment on 7 December 2023.

### Plant and environment measurements

The height and diameter of each plant were measured at the beginning and end of the experiment. Height was measured from ground level to the top of the apical shoot with a tape measure. The stem diameter was measured $\sim$10 cm above ground level using a digital caliper. The soil water content (%) was determined using the gravimetric method and expressed as volumetric content values (VWC), which were derived based on three oven-dried samples taken at the beginning of the experiment from fully saturated soil. The pots were weighed weekly (HW-60KGL scale, A&D, Japan), with the last weighing being performed in week 27 when there was enough evidence that the soil water conditions in all treatments were equal. Air temperature and relative humidity were measured at a 15-min interval using a EE181 sensor connected to a CR1000X data logger (Campbell Scientific Inc., USA) during the entire experiment. The vapour pressure deficit (VPD) was calculated with the Tetens equation. Once weekly over a 29-week period, the leaf stomatal conductance (to $\mathrm{H_{2}O}$ vapour) $g_{s}$ (mmol m$^{-2}$ s$^{-1}$) and net $\mathrm{CO_{2}}$ assimilation rate $A$ ($\mu$mol m$^{-2}$ s$^{-1}$) were measured on a cluster of needles (usually two fascicles from the upper part of each plant) on the 12 preselected plants using a GFS-3000 portable gas exchange and fluorescence system (Walz, Germany). The $\mathrm{CO_{2}}$ concentration, flow rate, temperature and relative humidity in the cuvette were set constant at 400 $\mu$mol mol$^{-1}$, 750 $\mu$mol air s$^{-1}$, 20 $^{\circ }$C and 60%, respectively. The light intensity in the cuvette was set at 1000 $\mu$mol photons m$^{-2}$ s$^{-1}$ PAR. Measurements were recorded after the values of $g_{s}$ and $A$ became stable. Gas exchange measurements were carried out between 09:00 and 12:00 h to minimize the effects of midday stomatal closure. The order in which individual plants were measured was chosen randomly every week. In week 10 of the experiment, we were unable to perform gas exchange measurements due to a power supply fault in the GFS-3000 system, resulting in missing data for that week. On two other occasions, a single plant was accidentally not measured (week 19 and week 22). In week 20, we carried out a one-off measurement of pre-dawn and midday leaf water potential ($\Psi _{L}$), on a single needle, in all 24 plants. The measurement was done before the LW plants were rewatered. The water potential was measured with a Scholander-type pressure chamber Model 1505D (PMS Instrument Company, USA).

Radial stem displacement was measured on 21 plants—seven per treatment group—using automatic dendrometers. Five plants were instrumented with high-precision (1 $\mu$m on radius) dual point dendrometers with a LoRaWAN wireless data logging system (DL-ZN2-002, Decentlab, Switzerland). These five plants included two from each water-stress treatment and one from the control group. Another 16 plants were equipped with lower-precision pivot dendrometers (0.1 mm on diameter; SDI-12 DPS-40, ICT, Australia). Data were recorded on four of those on a LoRaWAN data logger (IoT-Node, ICT, Australia) and using a CR1000X data logger for the remaining 12 plants. All measurements were recorded with a sampling period of 15 min or less. When gravimetric and gas exchange measurements were performed, the pivot dendrometers were removed from the plants and reinstalled afterwards. The initial position (height and azimuth) of each pivot dendrometer was recorded with a marker pen to minimize the error associated with repositioning. Dendrometers were installed at 10 cm above the root collar, except if a whorl was present at that height, in which case the installation was shifted 5 cm higher to avoid nodal swelling.

One of the plants in the control treatment, which was equipped with a point dendrometer and used for gas exchange measurements, experienced substantial damage caused by the screws securing the dendrometer. Signs of abnormal physiological responses, such as relatively low stomatal conductance, were observed as early as week 6. However, we continued to monitor the plant until the end of the experiment. During the last four measurement sessions, it consistently exhibited very low stomatal conductance and visible signs of needle turgor loss. Consequently, this plant (G1-1) was excluded from the analyses.

### Dendrometer data processing

Time series of stem radial displacement (SRD) recorded by the dual point dendrometers were processed to remove any variation between successive values (jumps hereafter) greater than 15 $\mu$m. The records of both dendrometers attached to the same device were averaged to compensate for any effect of leaning caused by the weight of the device. The SRD was processed to separate it into an irreversible expansion component corresponding to growth (GRO) and a reversible shrinkage component induced by the tree water deficit (TWD). This approach requires a conceptualization of the potential growth that may occur during shrinkage or re-expansion below a previous maximum in stem radius. We used the zero-growth model, which postulates that growth does not occur during periods of shrinkage (Zweifel et al. 2016). Under that assumption, the GRO component is calculated as the maximum value between the current SRD measurement and the GRO value calculated at the previous time step. It corresponds to the highest radius the stem has reached up to the time of the observation. The TWD component is derived as the difference between the GRO value and the SRD value at any time. It corresponds to the magnitude of the shrinkage currently experienced by the stem. In this study, the daily radial growth rate (iGRO) is calculated as the difference between two successive GRO values at midnight and is used to characterize growth activity. Daily TWD was calculated as the pre-dawn value, i.e., at approximately 05:00 h (Dietrich et al. 2018). It represents the residual stem shrinkage after nighttime refilling of water reserves and before any additional depletion induced by daytime transpiration. This is similar to using pre-dawn leaf water potential as the indicator of baseline water stress. Dendrometer data was processed by bespoke routines implemented in the Python programming language (https://www.python.org, v3.11).

Stem recovery from water stress, $R$, was defined using the trend of TWD following rehydration. It is the fraction of water deficit cumulated during a drought event that has been subsequently recovered after irrigation (Dietrich and Kahmen, 2019):


\begin{align*} &R = \left( 1 - \frac{\mathrm{TWD_i}}{\mathrm{TWD_0}} \right) \times 100,\end{align*}

where $i$ is the number of days after irrigation ($i=0$ on rewatering day, prior to rewatering). Recovery time was calculated as the number of days required to reach $R=100$%, at which point the stem water reserves were fully replenished. Recovery values and times were only determined for plants equipped with point dendrometers.

The data recorded by the pivot dendrometers were converted to daily SRD values equal to half the median diameter of the measurements recorded between 04:00 and 05:00 h on each day. Despite marking on the stem the initial sensor position, weekly handling of the plants and sensors introduced many signal artefacts. Those artefacts were inconsistent in terms of which plant would be affected, on which day, and what the magnitude of diameter variation would be, if any. No automated data processing could effectively and reliably remove these artefacts without introducing others. Consequently, the pivot data set was used to calculate the SRD trends for each treatment by fitting a GAMM (mgcv package; Wood, 2017). This processing averaged out the effects of sensor repositioning on stem diameter over time (see Figure S1 available as Supplementary Data at *Tree Physiology* Online). However, the dataset was not sufficiently robust to calculate GRO and TWD or to analyse stem movement at short time scales.

### Statistical analysis

We used Bayesian multilevel linear regression with a Gaussian likelihood to evaluate the effects of water stress treatments on $g_{s}$ and $A$ over time. For both $g_{s}$ and $A$, the models included the categorical predictor *treatment* (with three levels), the categorical predictor *week* (time-point with 29 levels) and their interaction (*treatment*  $\times$  *week*) to capture the time-varying effects of the treatments. The models also included two continuous covariates, vapour pressure deficit (VPD) and soil volumetric water content (VWC), which were centred and scaled to improve model convergence and interpretability. To account for the hierarchical structure of the data, we included random intercepts for individual plants (*tree_id*), nested within their respective genotypes (*clone_id*; two levels). The temporal structure of the data was modelled using an autoregressive term, allowing for the dependency of repeated measurements on the same plants over weeks. The intrinsic water-use efficiency (iWUE) was calculated as the ratio of $A$ to $g_{s}$, and expressed in $\mu$mol $\mathrm{CO_{2}}$ mol$^{-1}$  $\mathrm{H_{2}O}$. The iWUE had a highly skewed distribution and $\sim$9% of the data points had a value of zero. To assess the influence of VWC, VPD and their interaction on iWUE, we used a hurdle model with a lognormal distribution to account for the skewness and zero-inflation in the response variable. The hurdle component was modelled as a function of VWC, as this was clearly the main factor contributing to the complete closure of the stomata throughout our experiment.

Relative growth rates in terms of height ($RGR_{H}$) and diameter ($RGR_{D}$) were calculated as $RGR = [ln(y_{f}) - ln(y_{0})]/t$, where $y_{f}$ is the final height or diameter, $y_{0}$ is the initial height or diameter and $t$ is the length of the experiment in years. We tested the effect of water stress treatments on the relative growth rate using a multilevel linear model with *treatment* as a population-level predictor and random intercepts for the two genotypes (*clone_id*). Variance partitioning showed that variability was primarily expressed among individual plants, with a minimal and uncertain contribution of genotype; therefore, genotype-specific results were not reported.

All statistical analyses were conducted using R v.4.2.2 (R Core Team, 2022). Regression models were fit with the Bayesian inference programme Stan (Carpenter et al. 2017) using the brms package (Bürkner, 2017).

## Results

### Soil water content

The mean ($\pm$ SD) soil VWC in the control group during the course of the experiment was 34 $\pm$ 8% (Figure 1C). The decline in VWC followed a similar pattern in both water-stressed groups, with the EW and LW treatments reaching minimum values of 7% ($\pm$ 1%) and 4% ($\pm$ 1%) by the end of weeks 13 and 20, respectively. The EW group experienced low VWC levels (i.e., < 15%) for approximately 6 weeks, while the LW group was exposed to these conditions for 13 weeks, including a 7-week period during which VWC remained consistently below 7%.

### Leaf gas exchange response

**Figure 2:**
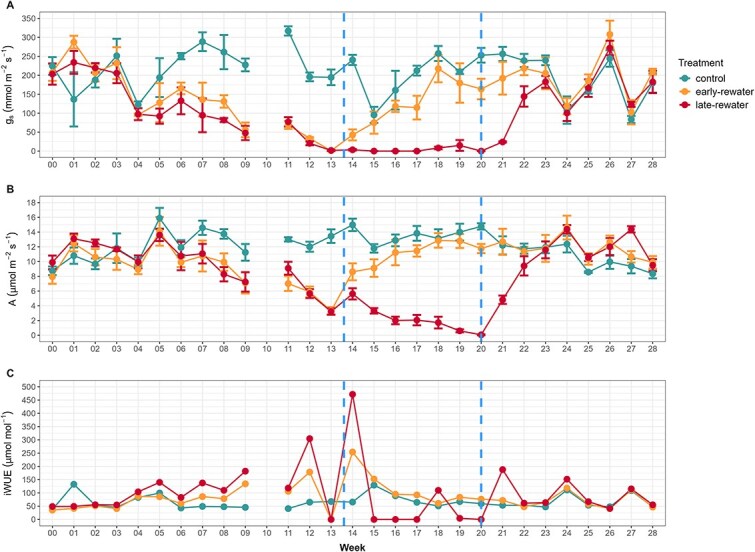
Temporal pattern of leaf stomatal conductance (A), photosynthetic assimilation rate (B) and intrinsic water-use efficiency (C) over a 29-week period. Points and error bars represent treatment group mean and its standard error value. Please note that in panel (C) only median values are shown. In panel (B), assimilation values recorded under severe water stress, when stomata are nearly or fully closed, should not be interpreted as reliable estimates of photosynthetic carbon assimilation, as measurements in this range are dominated by instrumental noise. Dashed vertical lines indicate first rewatering of the plants in the early- (middle of week 14) and late-rewater (beginning of week 21) treatments, respectively.

There  were no detectable differences in leaf gas exchange rates among treatments within the first 4 weeks of the experiment (Figure 2). During that period, water-stressed plants maintained a level of leaf function similar to that of watered plants. Subsequently, stomatal conductance and assimilation in water-stressed plants declined gradually. By the end of week 7, Bayesian multilevel model estimates (posterior means $\pm$ 95% credible intervals) indicated consistently lower $g_{s}$ in stressed plants (EW: 102 $\pm$ 99 mmol m$^{-2}$ s$^{-1}$; LW: 58 $\pm$ 103) compared with watered controls (288 $\pm$ 125). Assimilation showed a similar pattern, with lower $A$ in stressed plants (EW: 10 $\pm$ 4 $\mu$mol m$^{-2}$ s$^{-1}$; LW: 10 $\pm$ 4) relative to controls (12 $\pm$ 5). These differences in $g_{s}$ and $A$, with a similar pattern in both EW and LW, were maintained until the point when the groups were rewatered in weeks 14 and 21, respectively. The delayed but pronounced suppression of $g_{s}$ in the EW and LW plants became apparent in week 12, and during the measurements in the following week, we recorded complete stomatal closure in all plants (Figure 2A). However, after week 6, the decrease in $g_{s}$ in the water-stressed treatments was more pronounced than the reduction in $A$, leading to a substantial and consistent increase in leaf-scale iWUE (Figure 2C). During the period preceding complete stomatal closure, iWUE in the EW and LW groups was two to three times higher than in the control plants.

All water-stressed plants, including those in the LW group that experienced a 7-week period of minimal $g_{s}$ or closed stomata, started to recover gas exchange within approximately one week of rewatering (Figure 2A and B). Recovery of $g_{s}$ in LW plants during the first week after rewatering followed a slower trajectory than in EW plants, so the LW group required approximately one additional week ($\sim$2.5 vs 1.5 weeks) to reach levels comparable to the control. In contrast, $A$ recovered slightly more rapidly, both EW and LW plants attaining control-like rates within $\sim$1.5 weeks after rewatering. When recovery trajectories were compared over a longer post-rewatering period, the LW group showed a steeper overall increase in gas exchange than the EW group.

### Environmental modulation of intrinsic water-use efficiency

**Figure 3:**
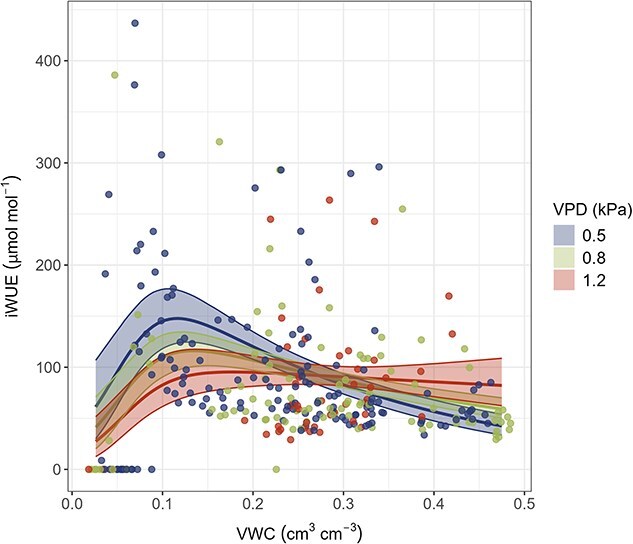
Response of leaf-scale iWUE to soil VWC and VPD. Curves represent the expected values of the modelled iWUE response across a gradient of VWC at three representative VPD levels. Shaded ribbons indicate 95% credible intervals around the estimates. Points represent the observed raw data and are grouped by VPD level.

According to estimated model parameters, iWUE was significantly influenced by an interaction between VWC and VPD (Table 1). Under well-watered conditions, iWUE was low, but progressively increased as soil moisture decreased (Figure 3). However, once the VWC decreased to $\sim$10%, the iWUE began to decline sharply due to strict stomatal control and subsequent complete closure. The relationship was further modulated by VPD, with the highest predicted iWUE values occurring under dry soil conditions in combination with relatively low atmospheric demand (i.e., VPD <0.8 kPa).

**Table 1:** Posterior summaries of population-level effects from the hurdle lognormal model predicting leaf-scale iWUE using soil VWC and VPD as predictors; please note both predictors were standardized. Estimates are based on posterior means and 95% credible intervals (CI).

Parameter	Estimate	SE	95% CI
*Lognormal part*			
Intercept	4.42	0.04	[4.34–4.50]
VWC	−0.26	0.05	[−0.35 to −0.17]
VPD	−0.05	0.04	[−0.13 to 0.02]
VWC:VPD	0.20	0.06	[0.09–0.31]
*Hurdle part*			
Intercept (hu)	−6.38	1.20	[−9.02 to −4.38]
VWC (hu)	−4.28	0.86	[−6.16 to −2.83]
Residual $\sigma$	0.59	0.03	[0.54–0.65]

### Radial growth response

**Figure 4:**
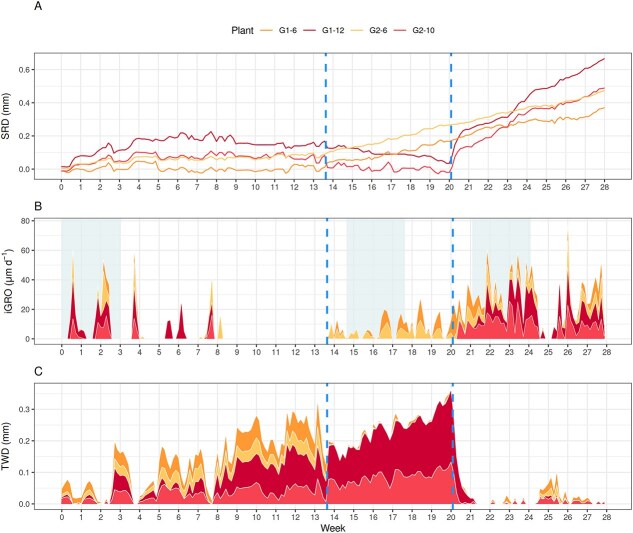
Changes in stem status over time for two plants released from water stress after 13.5 weeks (early-rewater, orange-tone colours) and two plants released after 20 weeks (late-rewater, red-tone colours). (A) Stem Radial Displacement (SRD); (B) daily growth increment (iGRO); (C) Tree Water Deficit (TWD). Area plots (B) and (C) are stacked, i.e., each curve is added to the one below. The shaded areas in panel (B) indicate the 3-week-long averaging windows for baseline growth rate, early-release growth and late-release growth (see Radial growth results).

The water-stressed trees equipped with point dendrometers ($n=4$) showed a phase of stem radial expansion for 4 weeks after soil irrigation stopped (Figure 4A) followed by a stable phase for another 4 weeks, during which stem radius remained relatively constant. Afterwards, all stems entered a phase of slow contraction. In the EW plants, contraction stopped in week 14 when the soil was rehydrated. In LW plants, stem contraction was accentuated from week 13 onward and continued for as long as the soil moisture deficit persisted, i.e., an additional 7 weeks. In both the EW and LW plants, the stem began to re-expand rapidly after rewatering. The overall trend in stem radial variation ($n=16$, see Figure S1 available as Supplementary data at *Tree Physiology* Online) showed a similar sequence of phases in response to stress and its release: (i) an initial phase of stem expansion tapering off; (ii) a latency phase with no expansion but also no clear contraction; and (iii) stem re-expansion following soil rehydration.

During the first 2 months of stress application, radial growth occurred episodically (Figure 4B). The magnitude and frequency of daily growth increments progressively declined during that period until complete cessation. Growth remained halted in both groups until rewatering, lasting 5 and 12 weeks, respectively. Growth resumed quickly after release in the EW group ($\bar{t}_{EW} = 3$ d), while a delay of 4–9 days was observed in the LW group ($\bar{t}_{LW} = 6.5$ days). In the EW group, the 3-week average daily radial growth rate increased from 2.2 $\pm$ 5.2 in early autumn to 3.2 $\pm$ 3.6 $\mu$m day$^{-1}$ in midwinter, after the release of water stress, to 5.1 $\pm$ 3.4 $\mu$m day$^{-1}$ in late winter. Each increase was significant ($P=0.008$, $W=640$ and $P=0.011$, $W=600$; Wilcoxon signed-rank test, two-sided). The mean daily temperature in the glasshouse for those 3-week periods was 17.6 $^{\circ }$C ($\bar{T}_{a}+4.8\,^{\circ }$C), 10.4 $^{\circ }$C ($\bar{T}_{a}+4.3\,^{\circ }$C) and 14.9 $^{\circ }$C ($\bar{T}_{a}+5.7\,^{\circ }$C), respectively, with $\bar{T}_{a}$ the mean air temperature outdoor. The increase in mean growth rate was associated with an increase in the number of days with active growth (‘growth coverage’ henceforth, given as a fraction of the averaging period). The growth coverage increased from 32% in early autumn to 62% after rewatering to 88% in late winter, showing that cambial activity became more regular and sustained over time. In the LW group, the mean daily growth rate increased from a baseline of 4.8 $\pm$ 6.7 to 11.6 $\pm$ 7.0 $\mu$m day$^{-1}$ after stress release. The increase was significant ($P<0.001$, $W=403$). The growth coverage also increased from 48% to 98%. The difference in growth rates between the EW and LW groups was not significant in the autumn, but it became significant in the late winter ($P<0.001$, $W=391$) with a mean growth rate 2.25 times higher in the LW group.

### Tree water deficit

**Figure 5:**
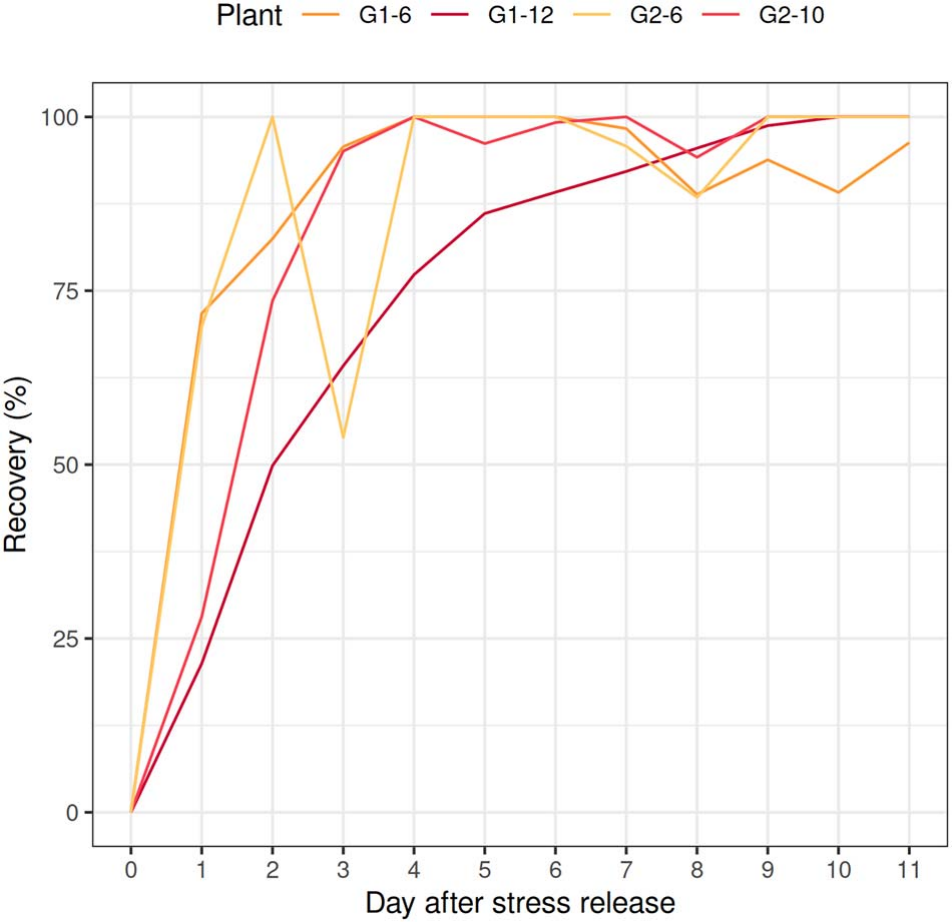
Percent recovery of pre-dawn tree water deficit following soil rewatering (early- and late-rewater plants are shown in orange and red tones, respectively).

The presence of tree water deficit was observed throughout the experiment in the water-stressed plants. Its magnitude did not increase in the first 2 months. However, non-zero TWD values became increasingly frequent over time and were recorded almost every day by the end of that period (Figure 4C). From week 8 to 14, TWD was non-zero on every day and for all trees with point dendrometers in both treatments. Despite a general increasing trend during that period, TWD fluctuations were best correlated with the variation in the atmospheric moisture deficit of the previous day. TWD was effectively reduced after days of low transpiration demand, such as the 2 days prior to stress release in the EW group. In the LW group, the effect of daily VPD fluctuations on TWD remained from week 14 to the end of week 20. However, the magnitude of the baseline TWD kept increasing over the period, to such levels that TWD remained high even on days with low transpiration demand. Complete recovery of stem water reserves occurred quickly after rewatering for both treatments (Figure 5). The EW trees recovered within 2 and 4 days after rewatering, with most of the recovery occurring on the first day (72% and 70%, respectively). The TWD magnitude was very low just before rewatering in the EW group (14.4 and 40.6 $\mu$m), which reduced the absolute amount of water deficit that those trees needed to refill when compared with the LW trees. The LW trees recovered from TWD values of 132.8 and 230.8 $\mu$m in 4 and 9 days, respectively, with only 28% and 21% of the total TWD recovered within the first day of stress release.

### Leaf water potential

**Figure 6:**
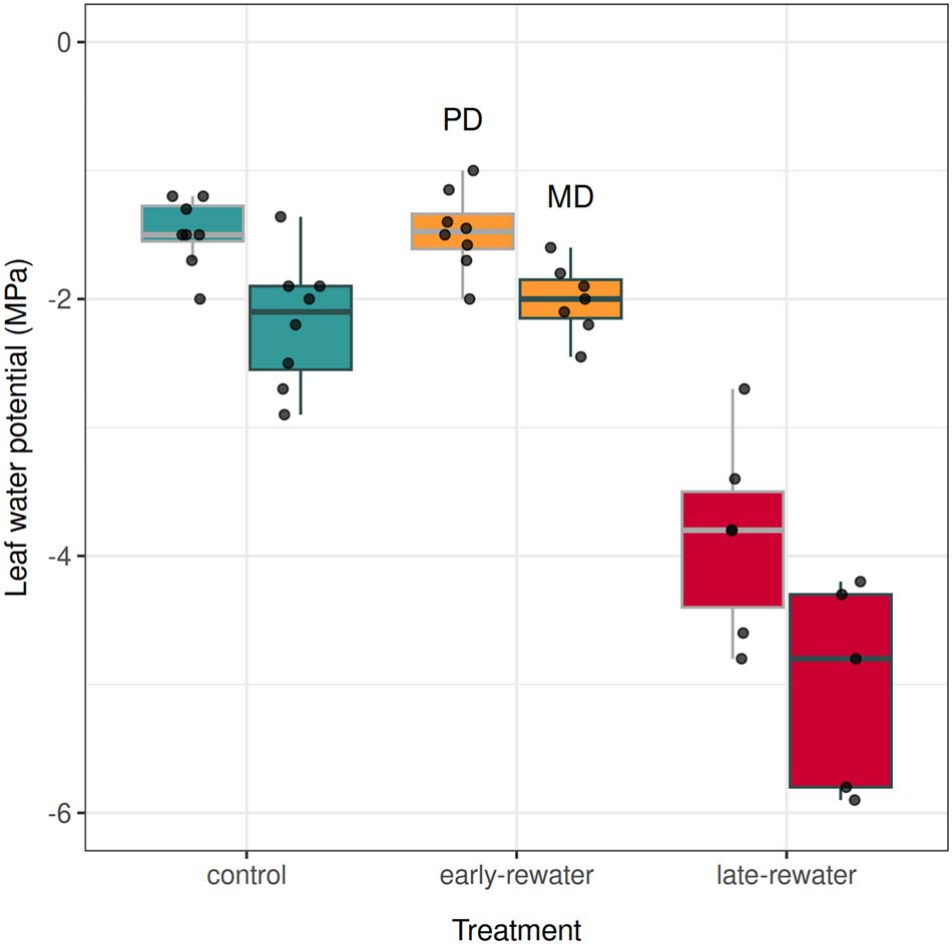
The PD and MD leaf water potential were measured on the day of late rewatering, before stress release. At the time of measurement, the late rewatering group had been under stress for 20 weeks, whereas the early rewatering group had returned to normal irrigation for 8 weeks. The boxes represent the interquartile range (IQR), with the horizontal line inside each box indicating the median value. Whiskers extend to the smallest and largest values within 1.5 times the IQR from the first and third quartiles, respectively. Gray jittered points represent individual raw data values.

By the time the LW plants were released from water stress, the leaf water potential had dropped to –3.85 $\pm$ 0.77 MPa pre-dawn ($n=6$) and –5.00 $\pm$ 0.81 MPa at midday ($n=5$). The differences between C and LW, and between EW and LW, were significant ($P<0.01$, Wilcoxon rank-sum test, two-sided) both at pre-dawn and midday. The needles of several LW plants (two plants at pre-dawn and three at midday) were embolized ($\Psi _{L}<-7$ MPa) prior to stress release. The early rewatered plants, which had been allowed to recover from water stress for 8 weeks, showed leaf water potential values that were not significantly different from those of the control group (Figure 6).

### Total height and diameter growth

**Figure 7:**
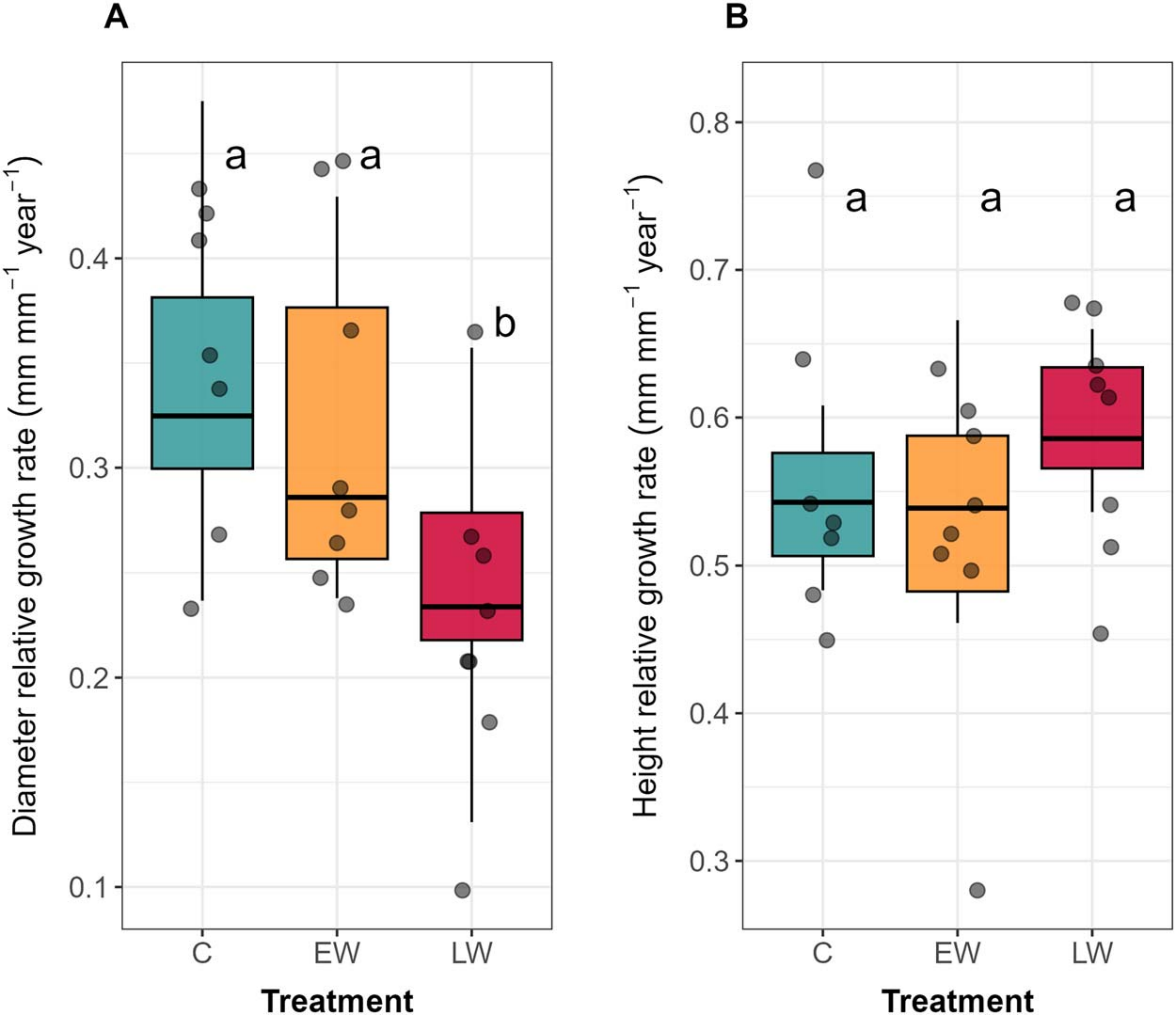
Stem diameter (A) and height (B) relative growth rate of plants grown under three treatments (C = control, EW = early-rewater and LW = late-rewater) over a 36-week period. The boxes represent the interquartile range (IQR), with the horizontal line inside each box indicating the median value. Whiskers extend to the smallest and largest values within 1.5 times the IQR from the first and third quartiles, respectively. Gray jittered points represent individual raw data values, illustrating the distribution within each treatment group. Different letters above boxes indicate significant differences between treatment means, as determined by the non-overlap of 95% credible intervals from a multilevel linear model. Note that the *y*-axis scale differs between plots.

Over the 36-week period, plants in both water-stressed groups exhibited lower relative stem diameter growth rates ($RGR_{D}$) than the unstressed control plants (Figure 7A). However, the differences in $RGR_{D}$ were small ($\overline{x}$  $\pm$ SE: 0.34 $\pm$ 0.03 mm mm$^{-1}$ year$^{-1}$ in C, versus 0.32 $\pm$ 0.03 in EW and 0.25 $\pm$ 0.02 in LW) and were marginally significant only for the late-rewater treatment, where, on average, $RGR_{D}$ was approximately 29% lower than in the control group. The relative height growth rates ($RGR_{H}$) of the plants in the three treatment groups were relatively similar (Figure 7B). The late-rewater plants had the highest mean $RGR_{H}$ (0.58 $\pm$ 0.02 mm mm$^{-1}$ year$^{-1}$), followed by the control (0.57 $\pm$ 0.04 mm mm$^{-1}$ year$^{-1}$) and early-rewater (0.52 $\pm$ 0.04 mm mm$^{-1}$ year$^{-1}$) groups.

## Discussion

Recovery from water stress in juvenile *P. radiata* depended on stress intensity, with prolonged stress causing slower recovery and a stronger decoupling of gas exchange and cambial growth. Cambial activity resumed faster and largely independently of leaf gas exchange recovery. Stress levels in the moderate (EW) and prolonged (LW) drought treatments corresponded to a transition from mild to moderate and from moderate to severe stress, respectively (sensu Ruehr et al. 2019). All stressed plants fully recovered in both gas exchange and growth. Although the recovery of $g_{s}$ in LW plants was delayed, no needle browning or other visible damage was observed. Even under severe drought, where LW plants experienced $\sim$4.5 months without water and $\sim$2 months of a neutral or negative carbon balance, cambial growth resumed rapidly once rewatered, largely driven by the recovery of stem water potential. Juvenile *P. radiata* thus exhibited high resilience (i.e., the capacity to re-establish pre-drought physiological functioning after stress release; sensu Lloret et al. 2011), with a prolonged water deficit followed by compensatory growth during recovery, provided the evaporative demand was not excessive. Although radial growth was impeded by stress and leaf function recovery was delayed, the whole-season aboveground biomass gain remained largely unaffected, even though LW plants had less than half the time under favourable conditions compared with the controls.

### Gas exchange dynamics during stress and recovery

During the drought phase, water-stressed plants maintained gas exchange at near-control levels for several weeks before it began to decline. This prolonged period of comparable functioning between stressed and unstressed plants in our experiment can be partially attributed to the glasshouse conditions, which were characterized by low evaporative demand, and to the potting mix we used, which had a high water-holding capacity. After $\sim$6 weeks, however, the whole-tree water deficit accumulated to a level where $g_{s}$ in the stressed plants decreased sharply, whereas the reduction in net carbon assimilation was less pronounced. This reflects the plants’ ability to maintain carbon assimilation while reducing water loss (Mitchell et al. 2014, Ruehr et al. 2019). Similar patterns of stomatal responses to declining water availability have been reported in earlier studies of gas exchange in *P. radiata* under drought (cf. Mitchell et al. 2014, Blackman et al. 2019, Rodríguez-Gamir et al. 2019). This species is known to exhibit a highly conservative water-regulation strategy (Mitchell et al. 2014, Sharma et al. 2025), in which strict stomatal control is strongly mediated by leaf-synthesized abscisic acid (Brodribb and McAdam, 2013). In our study, the reduced carbon assimilation in both stress treatments lasted $\sim$7 weeks, with the LW plants experiencing an additional 7-week period with a negative carbon balance. However, the carbon pool imbalances in water-stressed plants were likely partially moderated by increased water-use efficiency, which was notable in the period prior to complete stomatal closure (i.e., before week 13), caused by stomatal limitation preceding biochemical impairment of photosynthesis (Beer et al. 2009, Ruehr et al. 2019).

We found that leaf-scale intrinsic water-use efficiency had a clear hump-shaped response curve to declining soil volumetric water content, with a pronounced modulating effect of vapour pressure deficit. Specifically, iWUE increased as soil moisture declined from well-watered conditions to moderate drought levels (VWC $\sim$10–20%). However, once VWC fell below $\sim$10%, complete stomatal closure was recorded and iWUE decreased. The strongest iWUE response occurred under relatively low atmospheric demand (VPD < 0.8 kPa), highlighting that high VPD can constrain the potential of plants to increase water-use efficiency under drought (Novick et al. 2024). The reported interactive relationship between VPD and VWC and their effect on iWUE is based on observational data, as we did not control VPD levels in our experiment. Although our findings are in agreement with recent reports on the effect of VPD on plant water-use efficiency (cf. Yi et al. 2019, Li et al. 2023), the relationship reported for *P. radiata* needs to be further evaluated and verified under controlled conditions with varying levels of VPD, which would provide mechanistic insight into how VPD moderates the water-use efficiency capacity of this species under soil water deficit conditions.

Following rewatering, complete recovery of gas exchange was observed in all plants of both stress treatments, with substantial gains occurring already within the first week. However, we observed a clear lag between stomatal and photosynthetic recovery, as well as notable differences between the EW and LW plants. While the initial recovery of $g_{s}$ was slower in the LW group, comparison over a longer post-rewatering interval indicates a steeper overall recovery trajectory in LW than in EW, which may partly reflect the seasonal timing of stress release. Nevertheless, the prolonged-drought group took approximately one week longer than the moderate-drought group to restore stomatal conductance to control levels. Based on the initially delayed $g_{s}$ recovery in LW, we conclude that a drought-induced loss of xylem conductance likely occurred in these plants (Brodribb and Cochard, 2009, Ruehr et al. 2019). This is supported by the leaf water potential values obtained on the day before rewatering, where all LW plants displayed $\Psi _{L}$ values at or below the stem $\mathrm{P}_{50}$ value documented for the species (Blackman et al. 2019).

In contrast to $g_{s}$, $A$ reached control rates within $\sim$1.5 weeks for both stress treatments. The temporal divergence between $g_{s}$ and $A$ recovery has been noted in other studies before. In their review, Ruehr et al. (2019) found that leaf gas-exchange variables typically lag behind the recovery of leaf water potential after drought and that $A$ can often precede the recovery of $g_{s}$. Similarly, Brodribb and Cochard (2009) showed that plants suffering >50% loss of leaf hydraulic conductance had a much slower, $\Psi _{L}$-dependent gas-exchange recovery trajectory. Thus, the longer recovery time for $g_{s}$ in the LW plants likely reflects more severe xylem dysfunction requiring repair. Furthermore, the relatively flat recovery of $g_{s}$ in *P. radiata* plants, when exposed to prolonged drought, can also be attributed to stomatal reopening being constrained by the accumulation of abscisic acid (Brodribb and McAdam, 2013). Blackman et al. (2009) demonstrated that post-drought recovery of stomatal conductance and transpiration is strongly coupled to the recovery of leaf hydraulic conductance, and that abscisic acid signalling may constrain post-stress recovery.

It is important to note that our conclusions about the timing and rate of gas exchange recovery carry some uncertainty because measurements were made at weekly intervals and because seasonal changes may have influenced recovery trajectories. Nevertheless, our findings are consistent with the framework proposed by Ruehr et al. (2019) and earlier reports of recovery in *P. radiata* (cf. Brodribb and McAdam, 2013, Rodríguez-Gamir et al. 2019), confirming that gas-exchange recovery is dependent on drought intensity, and that once stomata start to reopen, photosynthesis rebounds faster than $g_{s}$, whose full recovery depended on the slower hydraulic supply and hormonal reset.

### Radial growth and stem water reserves dynamics

#### Stress phase

The responses of individual tree stems to soil dehydration showed three distinct phases, two when drought was not prolonged. During the first phase, which lasted up to 8 weeks, the radial growth process became more intermittent until it stopped. Water stress is known to inhibit plant growth (Muller et al. 2011, Tardieu et al. 2011, Krejza et al. 2021). At the cellular level, water stress affects both cell division (Kirkham et al. 1972, Sacks et al. 1997) and cell expansion (Vaganov et al. 2006) via turgor. Expansive growth relies on water intake and soluble sugars to generate turgor (Lockhart, 1965, Geitmann and Ortega, 2009). The growth process can be directly affected by a reduction in water intake caused by restricted water availability under drought stress (Daudet et al. 2005) or indirectly affected by a reduction in sugar concentration due to drought-induced stomatal closure, which reduces the assimilation rate (McDowell et al. 2008). Moreover, maintaining equivalent turgor under drought requires higher sugar concentrations and therefore a greater carbon demand when the water potential of adjacent tissues declines (Sala et al. 2012). The progressive shutdown of growth in this study resulted not from declining growth increments but from daily growth events becoming less frequent. It corresponds to a diminishing number of days with favourable conditions satisfying the constraints placed on turgor requirements by an increasingly limited water availability but still varying with daily atmospheric moisture deficit.

The slow onset of growth cessation can probably be attributed to the time required for soil VWC to reach levels low enough to impact plant water status. It would follow that stress intensity was low for the first few weeks of treatment. Since stomatal closure starts to be induced at relatively high water potential ($\sim$–0.8 MPa, Rodríguez-Gamir et al. 2019) and leaf physiology remained unperturbed for up to 4 weeks, the stem water potential was probably minimally affected. This would explain why cambial activity, typically very sensitive to water availability (Krejza et al. 2021, Li et al. 2021), took 8 weeks to stop entirely. Mitchell et al. (2014) recorded complete drought-induced growth cessation in *P. radiata* after a similar, though shorter, period (7 weeks). In our study, the progressive onset of cambial quiescence is unlikely to have been caused by phenology. It is known that cambial activity typically persists for *P. radiata* during autumn and wintertime, albeit at a reduced rate, under the temperate oceanic climate of the North Island of New Zealand (Tennent, 1986) and also in its native Californian habitat (MacDougal, 1938). Furthermore, the glasshouse environment provided even warmer winter conditions than would be encountered in those environments, with a mean daily temperature consistently remaining above the thermal threshold for cambial activity known for this species (8 $^{\circ }$C; MacDougal, 1938).

During the second phase of the water stress response, from 8 to 13 weeks without irrigation, tree stems consistently showed no daily growth and a residual nighttime tree water deficit. The absence of growth has been associated with water potential falling below a threshold (midday $\Psi _{L} < -1.4$ MPa), whereas non-zero TWD reflects a more general reduction in water availability (Zweifel et al. 2005). It is a common stress response caused by the limited capacity for refilling stem reserves at night through root uptake when soil water content is low (Zweifel and Häsler, 2001). It could indicate a partial recruitment of stem water reserves to supplement soil water uptake in order to meet the transpirative demand. This depletion of stem water reserves occurred despite the progressive stomatal closure and the relative reduction in transpiration losses. Since both stems and leaves displayed partial responses concurrently, we speculate that the TWD increase was driven by a general decrease in water potential across plant compartments. Low leaf water potential is associated with drought-induced stomatal closure (Mitchell et al. 2013, Rodríguez-Gamir et al. 2019), whereas a decreasing stem water potential is associated with an increasing TWD (Dietrich et al. 2018).

While TWD was non-zero throughout the second phase of stress, it remained tightly coupled with daily fluctuations of atmospheric moisture deficit. This behaviour can be explained by a limited night-time refilling of stem water reserves. Refilling is insufficient to eliminate TWD over the course of a single night but still occurs, as indicated by the absence of TWD build-up and a good correlation with VPD on a 1-day lag. This behaviour was evidenced by the sudden reduction in TWD, i.e., a reconstitution of stem water reserves, after 2 days of low VPD immediately before rewatering in the EW plants. We hypothesize that the second phase of the stem response to stress is an intermediate stage where the plants are gradually transitioning from normal to impaired functioning. This stage coincides with the progressive decline of leaf function. During that intermediate stage, the response appeared to be adequate in mitigating the reduced water availability in that stem water reserves were mobilized without being depleted.

In the third phase of the stress response during prolonged drought, there was a build-up in TWD, showing a clear increasing trend. It is associated with continuous reserve depletion and insufficient night refilling. That phase coincided with complete stomatal closure. This suggests that after mitigation of water losses has peaked in the leaf organs and can no longer buffer against stress, stem water reserves become strongly recruited. The maximum TWD in the LW treatment (125–225 $\mu$m) fell within the range of typical maximum TWD values in drought-stressed mature trees (75–200 $\mu$m in angiosperm spp., 400–600 $\mu$m in conifer spp.; Dietrich et al. 2018). However, the maximum TWD is notably high considering the juvenile status and the small diameter of the studied plants. For instance, the maximum TWD was close to or even exceeded the full width of the conducting phloem in juvenile *P. radiata*, which ranges from 100 to 180 $\mu$m (Gómez-Gallego et al. 2025). Given that the volume of deficit water exceeded the physical volume of the phloem, xylem water reserves must have been recruited as well. This is consistent with the expected pattern in the relative contribution of phloem and xylem tissues to stem shrinkage and hydraulic capacitance, shifting from mostly phloem-driven under well-watered conditions (Zweifel et al. 2005, Pfautsch et al. 2015) to composite phloem–xylem elastic contractions under drought (Steppe et al. 2015).

Stress severity has a major impact on the recovery trajectory, particularly on the recovery time and carbon costs (Ruehr et al. 2019). The notion of severity hinges on critical stress levels that induce transitions in the stress-recovery mechanisms. In the EW treatment, i.e., by the end of the second phase of stress, plants were very likely approaching the onset of hydraulic impairment and the transition to moderate stress. This is supported by growth ceasing 35 days prior to that point, a response that in this species occurs when $\Psi _{L}$ falls below –1.4 MPa (Mitchell et al. 2014). It is also supported by $\Psi _{L}$ stabilizing between –2 and –2.5 MPa after complete stomatal closure (Brodribb and McAdam, 2013). The existence of this stable phase is further corroborated by the lack of build-up in TWD and a stable stem $\Psi$. Individuals in the LW treatment likely reached the second critical stress threshold, characterized by the onset of hydraulic failure and resulting in irreversible structural damage. By the end of the prolonged stress period, either $\Psi _{L}$ had reached values equal to or below the stem $\mathrm{P}_{50}$ (–4.1 MPa; Blackman et al. 2019) or embolism had already occurred. Based on the vulnerability segmentation theory (Bouche et al. 2016), i.e., that distal organs hydraulically fail first (Brodribb and Cochard, 2009, Bartlett et al. 2016), we speculate that the stem hydraulic system was at the onset of embolism formation. Until the end of the third phase of stress, stem $\Psi$ continued to decrease, as indicated by increasing TWD values (Dietrich et al. 2018), whereas TWD would become unresponsive to further decreases in water potential once xylem conduits became embolized. Circumstantially, the prolonged stress period (140 days) corresponded closely to the dry-down period (164 days) recorded by Blackman et al. (2019) for the same species, with a similar seasonal shift towards winter progressively reducing transpirative demand.

#### Recovery phase

Tree water deficit recovered within a few days as previously observed after drought events (Dietrich and Kahmen, 2019, Rehschuh and Ruehr, 2022). The mean recovery time doubled from moderate to severe water stress. Although the difference may not be significant due to the sample size, the increase is consistent with a higher deficit reached under prolonged stress. The nonlinear kinetics of TWD recovery were typical of a hydraulic capacitor recharging passively. Recovery of TWD was initially rapid when the water potential difference between the rehydrated soil and stem tissues was largest, then slowed exponentially as stem water potential rose and the driving gradient decreased. The lower initial recovery rate in trees subjected to prolonged stress might have been due to the additional recruitment of xylem water reserves and a lower composite hydraulic capacitance compared with that of the phloem alone. Overall, the recovery of TWD is concomitant with the increase in stem water potential as rehydration proceeds. Stem refilling starting immediately after soil rehydration indicates that the water uptake function was intact and fine roots were not damaged. It must be noted that the reconstitution of stem water reserves proceeded at a faster rate than the recovery of leaf function. In a similar (but less efficient) manner to leaf shedding (Gauthey et al. 2022), the residual stomatal closure in leaves could benefit the reconstitution of stem water reserves by prioritizing water transport to them over transpiration losses. This behaviour could be characteristic of species in which abscisic acid imprinting makes stomatal closure persist even after leaf water potential has returned to high (i.e., less negative) values (Brodribb and McAdam, 2013).

The radial growth recovery pattern after drought release is still poorly described (Ruehr et al. 2019). Previous studies show a wide range of responses, from delayed recovery and long-term negative legacy growth effects (Hesse et al. 2023) to overcompensating growth (O’Brien et al. 2017). In this study, *P. radiata* showed a rapid recovery, with signs of stem re-expansion within 2–9 days of soil rehydration. The time scale of growth recovery is comparable to that of *Pinus sylvestris* L. (6 days) after a prolonged drought-heat event (Rehschuh and Ruehr, 2022). We speculate that growth activity followed stem water potential relaxation after rehydration to levels no longer limiting cell division and enlargement processes. This would imply that the cambial activity response to water stress is primarily driven by water availability to the meristem (Daudet et al. 2005). In contrast to the delayed recovery of leaf function, it did not show any sign of imprinting. The observed asymmetry between the kinetics of stem and leaf recovery indicates that recovery is not a process integrated at the organism level or, at least, that the integration is loose enough for organs to follow independent recovery trajectories in the short term. The rapid recovery of cambial activity that we observed has additional implications. Firstly, the cambium was not directly damaged by the build-up of negative pressure in the xylem. Water stress can induce such damage (Li et al. 2016), although this remains unlikely because the vascular cambium is among the most resistant tissues to water stress (Barigah et al. 2013). Secondly, the water-supply pathways to the meristem were not fully and irreversibly compromised as a result of drought.

To a large extent, radial growth was promoted after the release of drought stress. The mean growth rate was 45% higher after a moderate drought and 140% higher after a prolonged drought than the baseline activity. In both cases, the higher growth rates mainly resulted from an increase in the number of active growth days rather than from an increase in the magnitude of daily growth increments. The increased recovery growth rates beyond pre-stress levels and the apparent dose–response relationship between growth intensity and stress severity suggest a post-stress compensation mechanism (sensu Gessler et al. 2020). Post-stress growth rates higher than pre-stress ones have been observed previously (O’Brien et al. 2017, Rehschuh and Ruehr, 2022). In this study, seasonal effects might have influenced the magnitude of compensatory growth observed in *P. radiata*. Compensatory growth may have been inhibited in the EW group, released from stress at the coldest time of the year and enhanced in the LW group, released from stress at a warmer time. Overall, compensatory growth appeared to be approximately twice as high as baseline growth. It is also possible that phenology, which typically involves a late autumnal growth reduction, may have led to an underestimation of baseline, unstressed growth activity (defined here as that observed in April). If such a decline occurred, it was unlikely to have been induced by thermal constraints on cambial activity, as the mean daily temperature was 17.5 $^{\circ }$C, well above both the seasonal average and the critical threshold for cambial quiescence. Future controlled-environment experiments manipulating both drought intensity and seasonal conditions (e.g., temperature and photoperiod), and including more replicates, would allow for separating how stress intensity, phenology and individual traits contribute to recovery growth rates. Such experiments could also include measurements of root growth and root:shoot ratio to account for the effects of drought-induced allocation shifts (Rehschuh et al. 2022) on post-stress stem growth rates.

Even though radial growth appeared tightly coupled to local water status, its recovery must also have required an adequate carbon supply. We hypothesize that starch reserves were mobilized to support growth reactivation. *Pinus radiata* is known as a species able to mobilize those reserves to support growth under water stress, i.e., to operate at a low carbon safety margin (Mitchell et al. 2013, 2014). In addition, without active growth, those reserves can persist over long stress periods and thus be available upon stress release (Blackman et al. 2019). Alternatively, the carbon supply could have been provided by sugars accumulated in leaves during the stress period, a common stress response (Hartmann and Trumbore, 2016, Guo et al. 2021) and translocated upon resumption of normal phloem translocation after stress release. It is unlikely that fresh assimilates provided a sufficient supply to support growth recovery, as photosynthesis recovered at a lower rate. This is compounded by the fact that recovery growth rates that exceed growth rates in well-watered trees can occur independently of net carbon uptake (Rehschuh and Ruehr, 2022). Furthermore, isotope tracing studies have highlighted a prioritization of new carbon to storage and below-ground organs at the expense of above-ground sinks after drought release (Hikino et al. 2022, Rehschuh et al. 2022). Identifying the carbon sources and their relative contribution will be essential to understand whether rapid recovery and growth resilience are sustainable under chronic stress or if they make the plant vulnerable to fatigue by a progressive depletion of the reserves, as expected in drought response strategies reliant on a narrow carbon safety margin (Mitchell et al. 2014).

### Whole-season growth responses

Despite severe and prolonged soil water deficits, whole-season differences in growth rates between stressed and control plants were minor. Radial and height growth showed only limited or no reductions, respectively, in water-stressed treatments, suggesting a high degree of resilience in juvenile *P. radiata*. The overall growth pattern reflects reduced activity during stress, but strong compensatory recovery afterwards. This finding is consistent with O’Brien et al. (2017), who observed enhanced post-drought growth in seedlings of some tropical species they studied. In our study, late-rewater plants, despite experiencing the shortest window of favourable conditions and a 7-week period of high soil water deficit, attained overall increments similar to controls, highlighting the capacity for compensation of growth once soil moisture was restored (cf. Trugman et al. 2018, Gessler et al. 2020). However, radial growth was affected by the imposed water stress, with $RGR_{D}$ reduced the most in the late-rewater group. The rapid catch-up of radial increment during recovery also suggests that cambial activity responded flexibly to recovered water supply, consistent with accelerated post-stress growth reported in other fast-growing conifers (cf. Song et al. 2022). Furthermore, we found no differences in height growth rates among treatments. The direct causes for this response remain elusive, but we speculate that it may have been driven primarily by the timing of our experiment. The later stages of the experiment (i.e., the period after the LW group was released from stress) coincided with the austral late winter to spring period, when shoot elongation is the dominant growth process in trees (Tennent, 1986), whereas the earlier stages of the experiment when control trees could grow corresponded to the lowest levels of height growth activity. That combination of height and diameter growth patterns explain the very small differences in biomass accumulation despite drought effects.

Besides the aforementioned timing of the experiment, several mechanisms may explain why whole-season growth differences remained small overall. First, gas exchange recovered relatively quickly and fully after rewatering, restoring hydraulic and carbon assimilation functions to control-like levels. Second, Blackman et al. (2019) reported a non-declining NSC pool in *P. radiata* under prolonged drought stress, indicating that plants can avoid full carbon depletion, which could in turn enable rapid post-stress growth recovery. However, the opposite trend in NSC pools has also been reported, for example, Mitchell et al. (2013) observed that drought caused a significant depletion of whole-plant carbon reserves when plants were exposed to lethal drought. Thus, further detailed experiments are needed to elucidate underlying mechanisms and stress thresholds that shape carbohydrate reserve dynamics and determine the tipping points in species such as *P. radiata* that have a narrower carbon safety margin. Finally, stressed plants appear to have displayed high allocational responsiveness, reallocating carbohydrates to stem growth once water status became favourable again. These mechanisms together could explain why, despite the severe drought treatments, stressed plants maintained nearly comparable whole-season growth to controls. Importantly, we found no evidence of slow recovery or sustained growth suppression that would indicate widespread hydraulic impairment or whole-plant carbon depletion induced by imposed drought. Instead, the results indicate that juvenile *P. radiata* has functional traits that enable growth resilience through flexible responses, with compensatory stem growth mitigating the effects of prolonged drought in terms of aboveground biomass gain.

## Conclusions

Stress-induced growth reductions in the juvenile phase have important consequences for establishment and subsequent forest dynamics, because they influence interspecific interactions, long-term species composition, vulnerability to biotic stressors and overall forest productivity. Our findings provide a nuanced perspective on how carbon assimilation and growth in juvenile trees respond to water stress: species with a tight stomatal regulation (isohydric behaviour) that limits carbon supply and relies on stored reserves may nonetheless show substantial growth resilience following prolonged but non-lethal drought stress. The prompt resumption of cambial activity in the severely stressed plants—despite a prolonged period of near-zero carbon gain—suggests that these plants did not experience substantial carbon limitation immediately after rewatering, although the nature of the carbon supply to the lateral meristem has not been elucidated. These findings advance the understanding of the coordination of source and sink processes in juvenile trees and provide empirical evidence that can improve vegetation models’ representations of post-drought recovery in trees. However, our results were obtained under relatively low-to-moderate evaporative demand and from a single drought–rehydration cycle. Increased atmospheric aridity or chronic droughts could substantially impair the capacity for rapid growth resumption and/or compensatory growth by further constraining hydraulic recovery and causing carbon imbalances and limitation. Future work should therefore (i) quantify NSC dynamics during and after prolonged non-lethal drought, including resolving the relative contributions of recent and stored assimilates to recovery processes, (ii) experimentally manipulate VPD and repeat drought cycles to test the limits of compensatory growth and (iii) extend monitoring to multiseason outcomes (growth and survival) to assess longer-term demographic consequences and how these are shaped by recovery dynamics.

## Supplementary Material

Supplementary_material_tpag051

## Data Availability

The data supporting the findings of this study are available from the corresponding author upon request.
