# Supplementary material for: Water stress and recovery dynamics of physiological function and growth in juvenile Pinus radiata
Source: Tree Physiol. 2026 Jun 8;46(6):tpag051. doi: 10.1093/treephys/tpag051 (PMC13282971; doi:10.1093/treephys/tpag051)
Supplement: Supplementary_material_tpag051 [file supplementary_material_tpag051.zip › Firm-et-al_supplementary-data_tpag051.pdf]

Firm et al. "Water stress and recovery dynamics of physiological function and growth in juvenile *Pinus radiata*")

SUPPLEMENTARY DATA

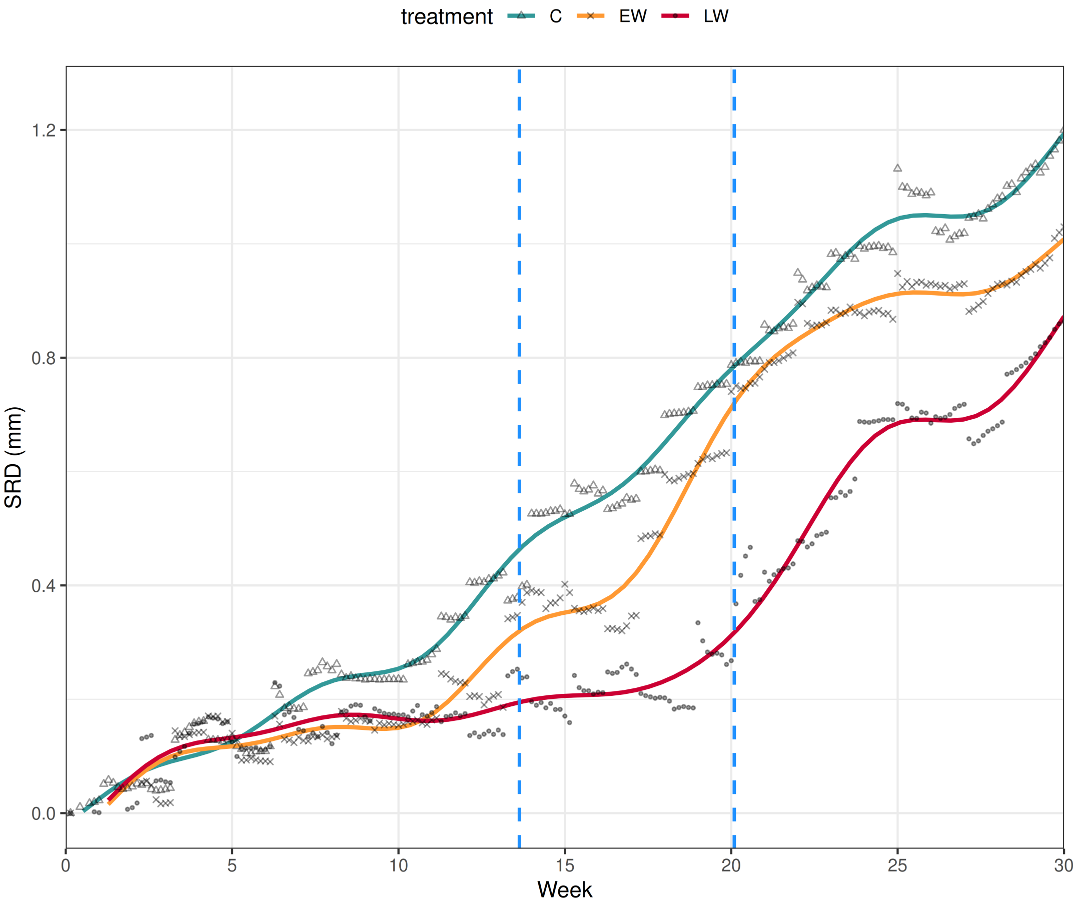

Fig. S1: Trends in stem radial displacement (SRD) measured with pivot dendrometers for each treatment group: control (C, n = 6) — watered regularly; early rewatering (EW, n = 5) — without soil irrigation for 13.5 weeks; late rewatering (LW, n = 5) — without soil irrigation for 20 weeks. Individual points indicate the mean daily SRD values for each treatment. The coloured line represents a GAMM fitted to individual SRD series grouped by treatment. Dashed blue lines indicate the timing of EW and LW soil rehydration events.

Figure S1 shows the overall pattern of stem radial expansion in response to continuous moderate and prolonged drought. Control plants, irrigated weekly, continued to grow throughout the monitoring period except for two lulls: one lasting about a month before the winter solstice and another after the spring equinox. Importantly, stems continued expanding at a sustained rate from week 12 to 20, i.e. during winter. Both EW and LW plants began to diverge from controls from week 6 onward, showing reduced expansion and indicating the onset of water stress effects on stem activity. In both treatments, stem re-expansion began around the time of stress release. Apparent pre-rehydration expansion likely reflects artificial jumps in SRD values caused by sensor handling (see Materials and Methods) and the effect of the smoothing window. Finally, both EW and LW plants showed sustained phases of expansion at rates higher than controls following soil rehydration, as well as a spring lull shared with control plants. Overall, the cumulative mean radial increment decreased with drought intensity, but the differences (0.9–1.2 mm) were relatively small and much less than the differences in the duration of periods when soil water was not withheld.
